# Medicine

**Published:** 1895-06

**Authors:** George Parker


### Progress of the Medical Sciences.

### MEDICINE.

Complaints are often justly made of the tendency to invoke with little or no proof some bacterial cause as the active agent in every disease; and on the other hand, a skilfully-argued paper recently appeared denying any bacterial origin for disease. We cannot afford, however, to neglect even a rational hypothesis which will account for the occurrence of many diseases in an organism so self-protected as the human body, though such hypotheses demand the most vigorous testing before they can be finally accepted. Why is a blow in ninety-nine cases out of a hundred followed by no harm to the tissues, and in the hundredth a cancerous growth ensues? Why do the kidneys deal with the multifarious results of metabolism, cope with the chills of every winter, protect the body from the effects of sudden exertion, and yet perhaps suddenly fail under the stress of some slight febrile attack? The organism which possesses the power of accommodating itself to changing circumstances in this peculiar degree; to increase or decrease of food, exercise, warmth, and moisture; which carries on its functions and maintains the thread of life under the most varying conditions, just as it maintains its uniform temperature in every climate,—this body suddenly loses in some one function its power of adaptation, and a breakdown occurs. Whence, then, comes in disease? Chills, overwork, want of nourishment do not of themselves account for all cases, though they may prepare the way for them. It is natural to expect that some exceptional poison against which the organism had not yet learned to protect itself, coming occasionally from the outside, would oftentimes be the cause. Now, we have found that certain organic bodies have remarkable power in producing changes in the human organism against which it frequently fails to defend itself—that they elaborate certain rare poisons which it neutralises with difficulty, while it easily copes with ordinary dirt and dust provided they do not contain these germs and these poisons. If the germs only broke up and digested the food material which is stored up in the body, it would be a mere struggle for subsistence between the host and the invaders; but, for some unknown reason, their mode of assimilating food differs from that of mere parasites, and inflicts severe and often fatal injuries on their hosts. Though their mere presence is rarely hurtful, they have a habit of predigesting their food as it lies outside their own bodies. Just as their hosts do, they produce during digestion various enzymes, albumoses, and alkaloids; but the host is unable to excrete quickly enough both these poisons and its own, and disease follows. It is conceivable that in time the host might adapt itself to them as parasites; but in the existing stage of things, it either renders itself uninhabitable for them or dies if it fails to do so. Either result is unfortunate for the invaders; but their descendants find a new home and time to multiply in a fresh individual, and the aim of Nature is accomplished.

Now, this not only explains, if true, the origin of very many otherwise inexplicable diseases, but, since it has been clearly proved that the introduction of such organisms into the system when all else is unaltered is regularly followed by certain morbid processes or diseases, we have good reason for inquiring in what cases do such causes really exist. We must avoid, on the one hand, attributing to microbes effects otherwise produced; and, on the other, overlooking the important co-operating causes without which they are powerless. Thus cholera germs produce no disease in some individuals where the flora of the digestive tract or other circumstances are adverse to them.

The etiology of the diseases of the nervous system was not long ago almost a blank space where men wrote "chills" and "idiopathic" in place of definite causes, although the pathology and classification were already carried to high perfection. It was known that some nerve-diseases occasionally followed fevers, and the connection of others with syphilis was much discussed; but of late great advance has been made. We might instance the light that has been thrown on some of these affections by the study of the lesions produced by the diphtheritic poison, and through the experimental study of others by the injection of various microbes and their toxins. The recent discussion at the Royal Medical and Chirurgical Society on the paralyses of syphilis—itself almost certainly a microbial affection—shows the stimulus and the new direction given by these researches. Jonathan Hutchinson dwelt on the types of neural affection produced in the early or second stage of syphilis as analogous to those occurring in ordinary fevers.[1] How the nerve-diseases of the tertiary stage, indeed, are produced is uncertain; but the interesting class of cases of paresis of both sensation and motion, the paraplegias, and the paralyses of single nerves—all of which are sometimes found during the acute secondary stage,—are curiously parallel to the affections following the acute exanthemata and the acute lesions of the eye and ear occurring in the same stage. Many of them are secondary to changes in the blood-vessels; and Hutchinson especially noticed their acute, rapid character and their amenability to treatment as contrasted with the otherwise similar lesions seen in the tertiary period.

Broadbent remarked on the symmetrical distribution of lesions in the secondary stage—their dependence on a disturbance of nutrition through the blood; while tertiary lesions he held to be produced by tissue-changes which had ceased to

[1] *Lancet*, 1895, vol. i., p. 677.

have any immediate relation to blood-changes. Gowers referred to the analogy of the diphtheritic organism and the poisons it excretes—so ably worked out by Sidney Martin—and would regard tabes and other tertiary lesions as caused by some chemical product of the syphilitic microbe left behind after it had disappeared from the blood. Hutchinson looks on them as due to a local implication of cell-structures which had led to cell-growth. Many cases of nerve-affections in early stages of syphilis were detailed in the course of the discussion, and the pathological evidence pointed to a mode of origination identical with that of similar lesions in the exanthemata. A leading article in the *British Medical Journal* of March 30th, 1895, refers, in connection with this subject, to the work of Vidal and Bezançon, who experimented on the effect of injections of streptococci, and produced myelitis in 6 per cent. of their cases. They had four instances of complete paraplegia, which in one case was ascending in type. There were diffuse degenerations of the cord and engorgement of the vessels; but the most careful search failed to show the microbes in the cord, though they were easily found in the blood. Hence the lesions were probably caused by a soluble poison formed elsewhere in the body. Many other organisms have been found capable of producing disseminated myelitis. The bacillus coli, under certain circumstances, is one of those offenders. Scalfati[1] confirms the view that syphilis directly produces at times acute meningo-myelitis as early as six months after the primary infection. This may be accompanied with complete sphincter paralysis, sensory disturbances, and marked trophic lesions such as bed-sores; but he does not think that these cases are easily influenced by treatment. The meningeal symptoms may be very slight; and Lamy, whose case was discussed by Hutchinson in the above debate, holds that the affection starts from the vessels, and that the veins are affected first and usually to a greater extent than the arteries. In short, a large amount of evidence is being brought forward that these nerve-diseases of the early stage of syphilis arise in the precise manner of those produced by known microbes. The relations of cord diseases to the distribution of, and lesions in, the vessels is the subject of another valuable paper by R. T. Williamson;[2] while Putnam[3] of Harvard gives an elaborate review of what is known as to the mode in which infectious processes produce the nerve-lesions which follow them. The latter points out that the spinal scleroses occur under such a variety of circumstances—as syphilis, heredity in Friedrich's disease, anæmia, and pellagra poisoning—as to suggest predisposing tendencies or a place of least resistance in the tissues which favours the actions of the special poison and renders the columns peculiarly vulnerable to many different foes. Yet, an infectious element cannot be

[1] *Riforma med.*, Jan. 14, 1895, quoted in *Brit. M. J.*, 1895, vol. i., Epitome, p. 21.
[2] *Med. Chron.*, N.S., vol. ii., 1895, p. 161 *et seq.* [3] *Am. J. M. Sc.*, vol. cix., 1895, p. 254.

ignored altogether. Thus he gives a case of disseminated sclerosis following malaria. Among other diseases in which an infectious origin is suspected are chorea, herpes, amputation neuritis (which is said to be rare in aseptic operations), poliomyelitis, and Landry's disease. Œttinger and Marinesco,[1] in discussing the latter disease, allow that the anatomical substratum is not always the same, but they show that it is often of infective origin. In their own case, indeed, they demonstrated a widespread streptococcal invasion; but it may occur in certain forms of intoxication, even alcoholic, just as much as from several kinds of micro-organisms.

James Taylor[2] gave yet another paper at the Royal Medical and Chirurgical Society on the scleroses produced by anæmia, in which he held that the frequency with which anæmia and sclerosis occurred together was too frequent to be accidental; while the symmetry of the sclerotic changes was against the view that they were due to hemorrhages. Gowers pointed out that their localisation was the same as that in lead, arsenic, and even in diphtheria poisoning. A similar affection is occasionally met with in diabetes; and this also chiefly occurs in the posterior columns. Whether the sclerosis in anæmia is dependent on any special poison or auto-intoxication is not yet clear, though a large number of cases have been brought forward and examined. An account of many such instances was collected by H. M. Bowman[3] and published in *Brain* last year, together with the details of a case under his care. The sclerosis was most marked in the posterior columns, but was found also in the anterior and lateral ones. Great changes in the vascular walls in the diseased area were observed; and Bowman, on the whole, considers that the anæmia was the origin and source of the degenerations. It has been suggested that all these cases were really pernicious anæmia, and that this disease is, in fact, one of toxic origin. But Taylor carefully guarded himself from calling his cases by that name; and Stockman[4] brings forward much evidence to show that that affection is merely a high degree of anæmia produced by many different causes and marked by numerous capillary hemorrhages. Still, there is some reason to believe in an intoxication occurring in chlorosis and other anæmias.

On the other hand, we may avoid such a view as insufficiently supported, and agree with Edinger that even the nerve-diseases which follow specific fevers are caused by exhaustion so great that "reconstructive metabolism fails to furnish the amount of restitution which the function of nerve and cell requires." Any disturbance of nutrition may, he says, affect certain areas sooner than others which are better supplied

[1] *Med. Week*, vol. iii., 1895, p. 85.
[2] *Brit. M. J.*, 1895, vol. i., p. 699. [3] *Brain*, vol. xvii., 1894, p. 198.
[4] *Brit. M. J.*, 1895, vol. i., p. 965.

with blood, and then the weakened tissue makes place for the surrounding tissues which grow into it.[1] There may be excess of function with only normal repair, or normal function with deficient repair. The former type is that of employment-neuroses, and also explains the greater frequency of tabes in men of active life than in women. It may, of course, be replied that poisons of external or internal origin are powerful in preventing repair, and often act in this way as well as by directly causing disintegration of tissue.

In view of this tendency to test everything in the light of some theory of infectious or microbial poison, it was certain that many efforts would be made to apply such a doctrine to acute rheumatism. Dr. A. Newsholme[2] supplies the latest survey of the subject. He points out that the disease occurs chiefly in winter and spring after a deficient rainfall, just as other fevers do. The mode of onset, the progress of the illness, and the curve of the temperature are analogous to theirs. The joint-lesions, the hyperpyrexia, endocarditis, the tendency to relapses are parallel phenomena in both. In liability to second attacks we are reminded of erysipelas. He considers that the want of infectiousness of rheumatism is due to the poison being buried in the joints and rarely escaping by any of the emunctory organs, as it does in typhoid or measles. The action of the salicylates is another point in favour of a microbial cause, but there is direct evidence that the poison clings about certain houses; and, again, that actual epidemics of the disease occur at intervals, and that these gradually spread from place to place over a country. He supposes the *materies morbi* to be a saprophytic organism which tends to take on parasitic habits in favourable circumstances, such as a scanty rainfall and a soil dried by a hot season. The endocarditis which is so frequent a complication is found in an increasing number of infectious diseases, gonorrhœa, erysipelas, tuberculosis, and pneumonia, as well as in typical exanthemata. Chorea, which, according to Osler, is more frequently followed by endocarditis than any other disease, is believed by many to be equally of zymotic origin, if indeed it is not due to the same cause as rheumatism, The preference of rheumatism for an injured joint is, indeed. explained by some experiments of Bouchard's. He found on injecting bacteria or administering lead to animals in whom a joint or other part of the tissues had been injured, that they could be found at the site of the lesion in excessive amount. But it must be confessed that though Leyden, Sacaze, and others claim to have isolated the germ of acute rheumatism, it has not been possible as yet to reproduce it by inoculation, and so to complete the fulfilment of the fundamental rules laid down by Koch. Sacaze, indeed, believes that staphylococci play, at

[1] *J. Nerv. & Ment. Dis.*, vol. xxii., 1895, p. 33.
[2] *Lancet*, 1895, vol. i., p. 589 *et seq.*

least, a considerable part in the causation of the disease, and that a wound of some kind is generally to be found which precedes the attack, and through which the germs have gained entrance.[1] Nor is he without proof that they are able to produce synovitis; but, then, other poisons do that also, and the whole argument is inconclusive. If staphylococci are present at all, it is possible that their functions are only preparatory, as in some experiments of Mosny and Marcano,[2] where rabbits in which a little of the staphylococcus toxin had been injected without effect died some time after from the induced virulence of the bacteria coli, which were enabled to cause peritonitis under the stimulus.

Before leaving the subject of bacterial action, we may refer to some most interesting researches on the cause of recovery and immunity in acute diseases by Cobbett and Melsome, conducted by means of experimentally-produced erysipelas in rabbits. They point out[3] that in all infectious fevers, under favourable circumstances, "at some period in the course of the disease a change takes place in the body of the patient which makes it a less favourable place for the microbes to live in than before; they consequently disappear, and recovery ensues. Moreover, for some time afterwards the patient is found to be less susceptible to the attack of the same microbe." Is, then, immunity due to the same cause as recovery? Now, in erysipelas the parts first affected recover while the disease is spreading elsewhere; and Cobbett and Melsome prove that these parts have attained a local immunity of a perfect character. The disease usually terminates before the entire body is attacked, hence there is reason to suppose a general immunity is produced; and this, too, they prove to be the case. Both the local and the less absolute general immunity are of short duration. Now, they argue that recovery and immunity are produced by the same cause—an inflammatory reaction. Thus, when erysipelas is spreading over the skin, we find a narrow outer zone—pale, œdematous, and full of cocci; within it is a red inflamed area, in which cocci are not to be found after twenty-four hours. The red zone spreads more rapidly than the pale, until reaction coincides with invasion and the disease is at an end. Moreover, this reddened area is found to be immune, and the part remains so for a short but definite time. If cocci are injected into it, a new inflammation takes place much more quickly than in any unprotected area. There is no pale zone formed. The cocci are at once destroyed and cannot be found, and the reaction subsides, having done its work of protection even before inflammation has commenced in a susceptible area. This rapid and intense reaction is less when a partial or incomplete immunity has been gained, such as is produced by an

[1] *Arch. gén. de méd.*, 1894, p. 513, quoted in *Am. J. M. Sc.*, vol. cix., 1895, p. 207.

[2] *Med. Week*, vol. ii., 1894, p. 595. [3] *J. Path. & Bacteriol.*, vol. iii., 1895, p. 39.

attack of erysipelas in another part of the body, or by the injection of the poison into the peritoneum. Thus the power of quickly and forcibly reacting is an important factor in immunity, local and general. It is acquired during the course of the disease, and is the cause of recovery. In other words, the duration of a fever depends on the time needed for the inflammatory action to catch up and destroy the growth of microbes and their products. In perfect immunity, there remains a power or habit of reaction so strong and rapid as to prevent any growth; and the less complete the immunity, the slower is the reaction. Whether this reaction is produced by the cells or fluids of the body is undetermined; but the distinction is not important for the object our authors had in view.

A more important point to be considered is how far this explanation of recovery and immunity applies to the various types of bacterial infection—the septicæmias, and the intoxicating processes as well as the local inflammatory affections. Professor A. E. Wright,[1] in the discussion at the Pathological Society on the pneumococcus, adopted this classification, and placed lobar pneumonia primarily under the last heading. The emigration of white corpuscles into the alveoli of the lungs is, he thinks, comparable to their accumulation into an abscess; but the crisis has no parallel in the course of an abscess. It is due, probably, to a secondary septicæmia with energetic leucocytosis. When the crisis occurs the white corpuscles and the pneumococci largely disappear from the blood, and, through phagocytosis or some other process, the activity of the latter is brought to an end.

A very similar result is met with in relapsing fever where the crisis is followed by turgidity of the spleen, into which both sets of corpuscles are carried from the blood. A weak point in this explanation, however, seems to be that pneumococci are not commonly found in the blood during an attack of pneumonia to any large extent. There is rather a resemblance to the intoxication produced in diphtheria, where the germs lie outside the body and only the toxins are absorbed. But even if the cocci are confined to the lung alveoli while their toxins circulate in the blood, we have no proof of anything which happens to them at the crisis which accounts for that change. Washbourn's paper on their part in that disease and the production of immunity was of great interest, but added very little to previously known facts. Nor did he agree with Wright's view of the crisis. He acknowledges that lobar pneumonia, like epidemic meningitis and several other diseases, is caused by Fraenkel's pneumococcus, which is capable of taking on pathogenic powers occasionally, just as the bacillus coli at the other end of the body does. Immunity for a short time could be produced by inoculation of animals with water-broth cultivations,

[1] *Brit. M. J.*, 1895, vol. i., p. 302.

and the serum of these animals was protective if introduced into others. Whether this serum contains an antitoxin, or whether it acts on the organisms themselves, is not clear; nor does Washbourn find it easy to obtain a trustworthy toxin from the microbes. The *Lancet*[1] quotes a summary of the results of inoculation obtained recently. The brothers Klemperer employed both serum from immunised animals and from convalescent patients, as well as sterilised cultivations, and they report a fall of temperature and rapid convalescence in a number of cases. Foa, Scolia, and Carbone obtained similar results with serum injections; and Lava obtained some improvement by injecting serum from animals actually suffering from pneumonia. A valuable study of the action of this organism in epidemic meningitis was given by Flexner and Barker in the *American Journal of the Medical Sciences*,[2] where a full account of the literature of the subject is to be found. As Flexner remarks, while the reports of many observers leave no doubt of the occurrence of Fraenkel's coccus in most outbreaks of this disease, nothing has been so far proved as to the manner in which it gains access to the brain and circulation—whether through the digestive or respiratory tracts, or through chance wounds; nor, indeed, as to the cause which renders many individuals suddenly susceptible together. Of late, too, the pneumococcus has been found in certain more or less benign types of endocarditis, pleurisy, and arthritis, and its presence instead of the more virulent streptococci is an indication for less active surgical treatment.

The reabsorption of the white corpuscles from the lungs during convalescence from pneumonia sets free so large an amount of nucleo-albumen that, according to A. E. Wright, the marvel is that we escape complete intravascular coagulation. However, it is rapidly broken down, and excreted as albumose and uric acid. This origin of uric acid from the nuclein of white corpuscles is important as throwing light on its production in gout and the uric acid diathesis. Uric acid has been commonly regarded as one of the products of metabolism on proteids, and possibly a relic of ancestral methods of this change. Haig and others laid down that it is normally produced and excreted in a fixed proportion to the urea of about one to thirty-three, and any decrease in this proportion meant a retention of uric acid in the body with serious results. Horbaczewski has, however, shown that uric acid is formed from the disintegration of leucocytes, quite irrespective of the albuminous food broken down into urea. Diseases where leucocytosis is present—such as pneumonia and leucocythæmia, or drugs which produce it, as pilocarpine and alcohol, or even bodily exercise—are followed by an increased excretion of uric acid, while the urea is unaffected. Moreover, after a meal leucocytosis occurs, with a

[1] 1895, vol. i., p. 236. [2] Vol. cvii., 1894, p. 155 *et seq.*

rise of uric acid in five or six hours; but the urea is only increased later on, after digestive metabolism has taken place. In fact, they come from quite different sources. Finally, Horbaczewski showed that the pulp of the spleen and other organs mixed with blood and kept at the body temperature with plenty of oxygen produces uric acid, and that this is derived from the nuclein present. We may add that it is not increased by albuminous diet to the same extent as urea, or much decreased by abstention from it. Von Jaksch's results of the examination of the blood largely corroborate this view of the derivation of uric acid from the leucocytes. Thus he found an excess of uric acid in anæmia (with an excess of white corpuscles), in pleurisy, malignant disease, and pneumonia, as well as in kidney-disease where there was difficulty in elimination of it when formed. Levison's recent work on the uric acid diathesis gives a useful account of these investigations; and Vaughan Harley, in a paper on the subject,[1] discusses the treatment of uric acid gravel under (1) aids to the solubility of uric acid, (2) prevention of its excessive formation. He recommends for the first object piperazine given with alkalies, plenty of salines and vegetables, with a dose of alkali at night. For the second, he limits the amount of meat (as that indirectly, to some extent, increases the uric acid) and forbids alcohol and excessive exercise, and administers quinine and arsenic to check leucocytosis. In gout Levison warns us against soda, lithia, and piperazine in acute stages, and adopts a mixed diet with moderate meals and without alcohol as best suited to this theory of the production of uric acid. It is evident that these new conceptions open a wide field for careful investigation and revolutionise accepted teachings.

George Parker.

